# Electroencephalogram-based time-frequency analysis for Alzheimer’s disease detection using machine learning

**DOI:** 10.14440/jbm.2025.0069

**Published:** 2024-11-26

**Authors:** Sérgio Daniel Rodrigues, Pedro Miguel Rodrigues

**Affiliations:** Centre for Biotechnology and Fine Chemistry- Associated Laboratory, Faculty of Biotechnology, Catholic University of Portugal, Rua Diogo Botelho 1327, Porto 4169-005, Portugal

**Keywords:** Discrimination, Electroencephalogram, Mild cognitive impairment, Alzheimer’s disease

## Abstract

**Background:**

Alzheimer’s disease (AD) is the most common form of dementia. The lack of effective prevention or cure makes AD a significant concern, as it is a progressive disease with symptoms that worsen over time.

**Objective:**

The aim of this study is to develop an algorithm capable of differentiating between patients with early-stage AD (mild cognitive impairment [MCI]), moderate AD, and healthy controls (C) using electroencephalogram (EEG) signals.

**Methods:**

A publicly available EEG database was utilized, with seven EEG recordings selected from each study group (MCI, AD, and C) to ensure a balanced dataset. For each 1-s segment of EEG data, 43 time-frequency features were computed. These features were then compressed over time using 10 statistical measures. Subsequently, 15 classifiers were employed to distinguish between paired groups using a 7-fold cross-validation.

**Results:**

The strategy yielded better results than state-of-the-art methods, achieving a 100% accuracy in both C versus MCI and C versus AD binary classifications. This improvement translated to a 2% increase in accuracy for C versus MCI and a 4% increase for C versus AD, despite a 1.2% decrease in performance for AD versus MCI. In addition, the proposed method outperformed prior work on the same database by 4.8% for the AD versus MCI comparison.

**Conclusion:**

The present study highlights the potential of EEG as a promising tool for early AD diagnosis. Nevertheless, a more extensive database should be used to enhance the generalizability of the results in future work.

## 1. Introduction

Alzheimer’s disease (AD) is the most common form of dementia. Individuals over the age of 65 are at an increased risk of developing AD, as aging is the most significant risk factor for the disease.^1^ Western countries, where a considerable increase in average life expectancy is expected, are projected to experience a high prevalence of AD.^2^ There is currently no cure for this disease, and early diagnosis is crucial, as AD is a progressive condition with symptoms that worsen over time. Diagnosing AD in its early stages can be challenging because its symptoms are often similar to those of other conditions, such as normal aging.^3^ AD progresses slowly and affects most areas of the brain, impairing memory, thinking, judgment, language and problem-solving abilities, personality, and movement.^4^

AD progresses through four main stages, beginning with mild cognitive impairment (MCI), a pre-dementia phase in which cognitive deficits, particularly in memory, are subtle, and daily life remains largely unaffected. First defined in 1988, 5,6 MCI is considered a transitional stage between normal aging and AD. Although MCI increases the risk of developing AD, only 10%–15% of individuals with MCI progress to AD each year,^7^ with the cognition in some individuals either returning to normal or remaining stable.^4^ As AD progresses, it advances into the mild stage, where memory lapses and difficulties with complex tasks become more noticeable, although individuals can still function with some independence. The moderate stage follows, characterized by more significant cognitive decline, confusion, and increased dependence on others for daily activities. Behavioral changes may also emerge during this stage.^8^-^10^ In the final, advanced stage of AD, patients experience severe cognitive and physical impairments, losing the ability to communicate effectively or perform basic daily tasks. Full-time care and constant monitoring become necessary as the disease progresses.^8^-^10^

Early diagnosis of AD significantly impacts a patient’s cognitive function and is essential for implementing effective treatments to improve quality of life. MCI is particularly crucial in this process, as it serves as a key indicator for the potential development of AD. However, in clinical practice, MCI and AD diagnoses are primarily based on clinical assessments that evaluate cognitive function and functional status according to criteria established by the National Institute of Neurological and Communicative Disorders and Stroke and the Alzheimer’s Disease and Related Disorders Association (NINCDS-ADRDA).^11^-^13^ Despite using these standardized criteria, the accuracy of MCI diagnosis remains suboptimal, with accuracy rates of around 75%. This limitation underscores the need for the development of new diagnostic methods to improve AD detection accuracy, especially during the MCI stage.^14^

The brain generates electrical activity that can be recorded using electroencephalogram (EEG) signals.^15^ As a supplementary diagnostic tool, EEG provides valuable insights into the brain’s electrical activity and functional patterns.^16^ Since AD affects most areas of the brain, EEG can reveal both structural and functional deficiencies associated with the disease at different stages of its progression.^17^,^18^ By capturing neural signals, EEG allows for a better understanding of how the brain functions and responds in AD.

For decades, EEG has been a valuable diagnostic tool for dementia, recording spontaneous electrical brain activity with high resolution through scalp electrodes. Since AD affects neural activity, EEG can assist in its identification.^19^ EEG is widely used in clinical settings due to its affordability, non-invasiveness, portability, and speed. EEG signals are typically divided into frequency bands: delta (1–4 Hz, δ), theta (4–8 Hz, θ), alpha (8–13 Hz, α), beta (13–30 Hz, β), and gamma (30–40 Hz, γ).^19^ AD notably alters EEG power, increasing activity in low frequencies (δ and θ) and decreasing activity in high frequencies (α and β).^20^,^21^ These changes are linked to the degeneration of cholinergic synapses in the Meynert nucleus, which disrupts acetylcholine synthesis, leading to impaired synaptic synchronization and slower EEG waves.^20^,^21^

In this study, we leveraged machine-learning (ML)-based EEG signal analysis approaches to differentiate between the MCI stage, moderate-stage AD, and healthy controls. Our primary objectives are as follows:

- To introduce the synergistic use of 43 linear and non-linear time-frequency features to characterize AD activity throughout its progression.
- To enhance the assessment of distinguishing between binary classifications of study groups (MCI, AD, and healthy controls) by analyzing the synergistic impact of the extracted features with 15 ML classifiers.

## 2. Materials and methods

**Figure 1:**
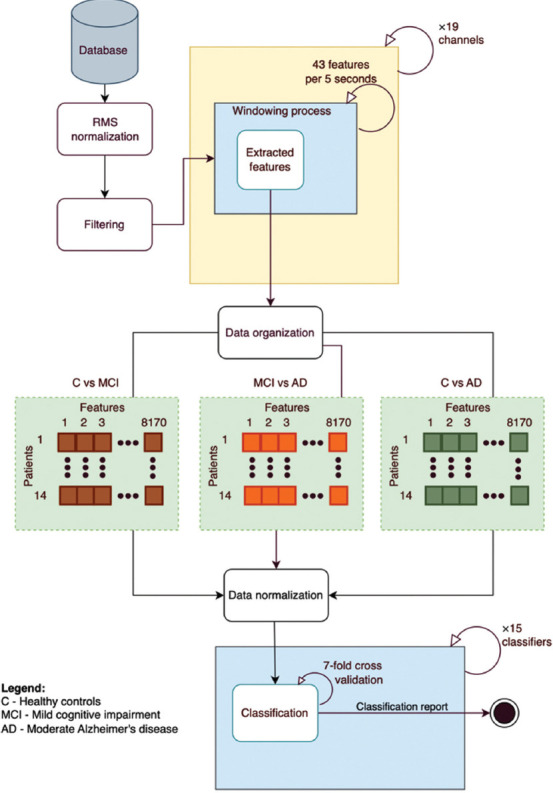
Electroencephalogram processing workflow diagram.

The methodology used in this study is illustrated in Figure 1 and is divided into three phases: (i) database acquisition, (ii) time-series analysis and feature extraction, and (iii) ML classification. All stages and analyses were developed in a Python (version 3.9.12, Python Software Foundation, Wilmington, Delaware, EUA) environment on a Mac mini equipped with an M2 chip, featuring an 8-core central processing unit, a 10-core graphics processing unit, 8 GB of random-access memory, and a 256 GB solid-state drive.

### 2.1. The database

The database was retrieved from a public repository (https://doi.org/10.6084/m9.figshare.5450293.v1). Further details can be found in the study by Cejnek *et al*.^22^ The database includes data from seven individuals with MCI, 59 patients with moderate AD, and 102 healthy controls, all diagnosed according to the NINCDS-ADRDA Alzheimer’s criteria. The selection criteria for AD and MCI patients included: (i) cognitive impairment, measured by Mini-Mental State Exam scores ranging from 10 to 19; (ii) clinical history and examination; (iii) neuroimaging through multi-slice computed tomography scans to assess hippocampal atrophy; (iv) blood tests; and (v) cerebrospinal fluid biomarkers and functional assessments. Given that the database contains only seven MCI patients, for a balanced dataset, we randomly selected seven healthy controls and seven AD patients. EEG signals were recorded from patients with MCI and AD while they were resting with their eyes closed and without medication, at a sampling rate of 256 Hz. Healthy controls were also recorded under the same conditions, but their signals were sampled at 200 Hz. To ensure consistency across the dataset, EEG data from the control group were resampled to 256 Hz. A spectral analysis was performed to compare the frequency content of the original and resampled signals, and no distortions related to artifacts or phase shifts were detected; the correlation coefficient was near 1, indicating that the resampling process was successful. The EEG recordings were made using an international 10–20 system with 19 channels. Each patient’s EEG data underwent channel-wise root-mean-square (RMS) normalization, followed by mean value removal. RMS normalization facilitates the comparison of EEG signals from different individuals and groups by accounting for variations in signal amplitude, independent of the recording duration. The mean removal centers the signal, which is a particularly important point because the time-frequency metrics extracted later assume that the data are zero-centered. Subsequently, a 5^th^-order Butterworth band-pass filter, with a frequency range of 1–40 Hz, was applied to each channel. The demographic characteristics of each group are presented in Table 1.

**Table 1:** Overview of the database demographics

Group	Number of subjects	Age mean ± SD	MMSE
C	102	72.2±5.3	N/A
MCI	7	67±7.6	N/A
AD	59	70.5±4.9	14.9±2.3

Abbreviations: AD: Alzheimer’s disease; C: Healthy controls; MCI: Mild cognitive impairment; MMSE: Mini-mental state examination; N/A: Not available.; SD: Standard deviation.

### 2.2. Time-series analysis and feature extraction

In a sliding window process, lasting one second, 43 metrics were extracted per channel for each study participant (Table 2 for more details). These metrics included entropy metrics,^23^-^25^ basic statistical measures,^26^,^27^ power spectral density (PSD) metrics,^28^-^30^ frequency domain metrics,31 as well as fractal dimension and complexity metrics.^20^,^26^,^32^ After the extraction process, the time-series data from each channel were compressed using 10 statistical functions: mean, median, minimum, maximum, standard deviation, variance, and the 25^th^, 50^th^, 75^th^, and 95^th^ quantiles.^33^ This compression reduces dimensionality while preserving the most critical information, simplifying analysis, and improving interpretability. It also retains essential data characteristics, including central tendency, dispersion, range, shape, and robustness.

**Table 2:** Description of the features

Metric name	Equation	Variable description
Entropy metrics		
Entropy sample	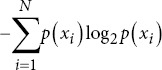	*p* *(x_i_*): Probability of the *i* -th state. *N*: The total number of samples
Permutation entropy	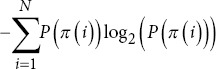	*P*(*π*(*i*)): Probability of the *i*-th permutation.
Spectral entropy	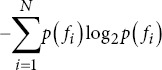	*p* *(f_i_*): Probability of the *i*-th frequency component.
Singular value decomposition entropy	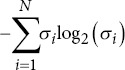	*σ_i_*: Singular values.
Approximate entropy	*ϕ_m_* *(r*)−*ϕ_m+1_*(*r*)	*ϕ_m_* *(r*): Function of embedding dimension *m* and tolerance *r*.
Sample entropy	−ln(*ϕ_m+1_*(*r*))	-
Basic statistical metrics		
Minimum	min *(x_1_, x_2_,…, x_N_*)	*x_i_*: The *i*-th sample in a dataset. *N*: The total number of samples.
Maximum	max *(x_1_, x_2_,…, x_N_*)	
Mean	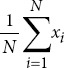	
Root mean square	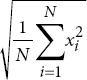	
Variance	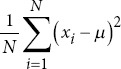	*μ*: Mean of the dataset.
Standard deviation	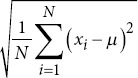	
Crest Factor	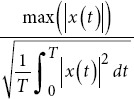	*T*: Time period.
Skewness	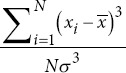	*σ*: Standard deviation.
Kurtosis	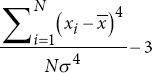	
Percentile 25	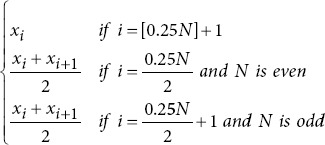	
Percentile 50	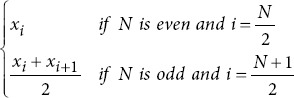	
Percentile 75	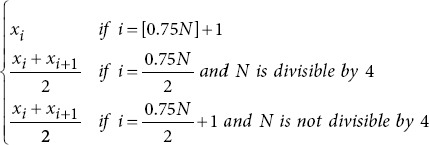	
Power spectral density metrics		
Power spectral density (*PSD*)	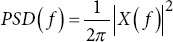	*X* *(f*): Signal’s Fourier transform
*PSD* peak	max *(PSD* *(f*))	
*PSD* peak-to-peak	max *(PSD* *(f*))−min *(PSD* *(f*))	
DELTA power	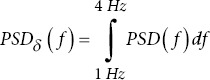	
THETA power	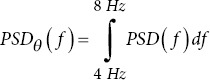	
ALPHA power	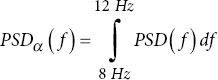	
BETA power	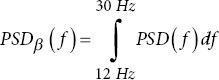	
GAMMA power	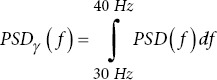	
*r*_1_	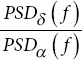	
*r*_2_	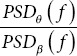	
*r*_3_	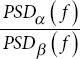	
Frequency domain metrics		
Maximum frequency	max *{f*∣*X* *(f*)≠0}:	*f*: Frequency of the signal. *X* *(f*): Signal’s Fourier transform
Sum of frequencies	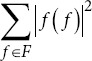	*F*: Set of all frequencies. |*f* *(f*)|^2^: Power at frequency *f*.
Mean frequency	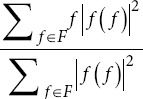	
Median frequency	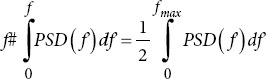	*f_max_*: Maximum frequency.
Peak frequency	argmax*_f∈F_* *| f* *(f*)|^2^	*f_p_*: Peak frequency.
Skewness frequency	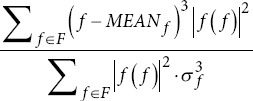	*MEAN_f_*: Mean frequency. *σ_f_*: Standard deviation of frequencies.
Kurtosis frequency	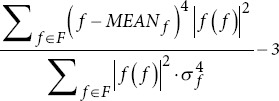	
Fractal dimension and complexity metrics		
Number of zero-crossings	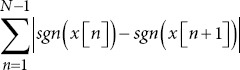	*sgn* *(x*): Sign function.
Katz fractal dimension	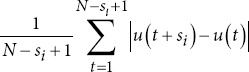	*s_i_*: Parameter in Katz fractal dimension formula. *u* *(t*): Function of time *t*.
Higuchi fractal dimension	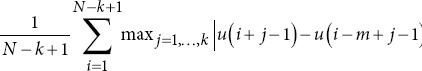	
Detrended fluctuation analysis	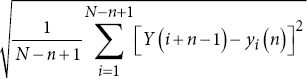	*Y* *(i*): Integrated time series. *y_i_* (*n*): Local trend for a window of size *n*.
Petrosian fractal dimension	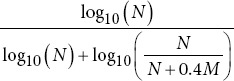	*M*: Number of extrema.
Hjorth mobility	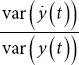	var *(y*(*t*)): Variance of *y* *(t*). : Variance of the first derivative of *y* *(t*).
Hjorth complexity	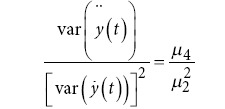	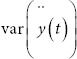 : Variance of the second derivative of *y* *(t*). *μ*_2_: Second moment (variance). *μ*_4_: Fourth moment.

### 2.3. Extracted EEG time-frequency metrics in the context of AD

The following items explain the context of each extracted EEG metric within the AD framework:

#### 2.3.1. Entropy metrics

- Sample entropy: Measures the complexity and irregularity of EEG signals. In AD, lower entropy values often indicate reduced complexity and more regular brain activity.^16^,^34^
- Permutation entropy: Quantifies the complexity of EEG signals by analyzing the order of values. In AD patients, lower values suggest decreased dynamic complexity.^16^,^35^
- Spectral entropy: Reflects the distribution of power across different frequency components. AD patients typically show lower spectral entropy, indicating a more predictable and less complex signal.^36^
- Singular value decomposition entropy: Assesses the complexity of EEG signals by analyzing singular values. Lower values in AD patients suggest reduced signal complexity.^37^
- Approximate entropy: Measures the regularity and predictability of EEG signals. AD patients often exhibit lower approximate entropy, indicating more regular brain activity.^16^,^34^,^38^
- Sample entropy (ln): Similar to approximate entropy but more consistent for shorter data lengths. Lower values in AD patients indicate reduced complexity and increased regularity.^34^

#### 2.3.2. Basic statistical metrics

- Minimum/maximum: The lowest and highest values in the EEG signal. These metrics help identify the range of brain activity in AD patients. Typically, AD patients have higher maxima and minima than healthy controls.^27^
- Mean: The average value of the EEG signal. Changes in mean values can indicate alterations in overall brain activity in AD patients.^39^
- RMS: It measures the magnitude of the EEG signal. Lower RMS values in AD patients can indicate reduced brain activity.^39^
- Variance/standard deviation: The metric measures the spread of EEG signal values around the mean. Lower values in AD patients suggest less variability in brain activity.^39^
- Crest factor: The ratio of the peak value to the RMS value, indicating the presence of spikes. Lower crest factors in AD patients suggest fewer spikes in brain activity.^39^
- Skewness: It measures the asymmetry of the EEG signal distribution. Changes in skewness can indicate alterations in brain activity patterns in AD patients.^39^
- Kurtosis: The metric measures the “tailedness” of the EEG signal distribution. Higher kurtosis in AD patients can indicate more extreme values in brain activity.^39^
- Percentiles (25^th^, 50^th^, 75^th^): These metrics help understand the distribution of brain activity in AD patients.^39^

#### 2.3.3. PSD metrics

- PSD peak/peak-to-peak: The highest power value and the difference between the highest and lowest power values. Changes in these metrics can indicate alterations in brain activity in AD patients.40.
- Delta/Theta/Alpha/Beta/Gamma power: The metric measures the power in specific frequency bands. AD patients typically show increased delta and theta power and decreased alpha and beta power.40
- Ratios (r_1_, r_2_, r_3_): Ratios of power in different frequency bands are used to identify changes in brain activity. Altered ratios in AD patients can indicate changes in brain function.40

#### 2.3.4. Frequency domain metrics

- Maximum frequency: The highest frequency component in the EEG signal. Lower maximum frequencies in AD patients suggest reduced brain activity.16
- Sum of frequencies: The total power across all frequencies. Changes in this metric can indicate alterations in overall brain activity in AD patients.16
- Mean/median frequency: The average and median frequency of the EEG signal. Lower values in AD patients suggest a shift toward lower-frequency brain activity.16
- Peak frequency: The frequency with the highest power. Changes in peak frequency can indicate alterations in brain activity patterns in AD patients.16
- Skewness/kurtosis frequency: The metric measures the asymmetry and “tailedness” of the frequency distribution. Changes in these metrics can indicate alterations in brain activity in AD patients.16

#### 2.3.5. Fractal dimension and complexity metrics

- Number of zero-crossings: The number of times the EEG signal crosses the zero line, indicating signal variability. Fewer zero-crossings in AD patients suggest reduced variability in brain activity.40
- Katz/Higuchi fractal dimension: The metric measures the complexity of the EEG signal by analyzing its fractal properties. Lower values in AD patients indicate reduced complexity.40
- Detrended fluctuation analysis: The method assesses the long-term correlations in the EEG signal. Altered values in AD patients suggest changes in brain activity patterns.40
- Petrosian fractal dimension: It measures the complexity of the EEG signal by analyzing the number of extrema. Lower values in AD patients indicate reduced complexity.40
- Hjorth mobility/complexity: The metric measures the variability and complexity of the EEG signal. Lower values in AD patients suggest reduced variability and complexity in brain activity.40

### 2.4. Data organization

The data were organized into matrices of 14 rows and 8170 columns for pairwise comparisons (C vs. MCI, AD vs. MCI, and C vs. AD). This format is compatible with Sci-learn ML models using Python DataFrame. The 14 rows correspond to individual patients, while the 8170 columns result from the analysis of 19 channels × 43 features × 10 data compressors. The data were normalized using min-max normalization41 for each group pair’s matrix columns (using all data from subjects within each binary group), as the distributions of the metrics are not significantly affected by outliers.

### 2.5. ML-based classification

The pairwise comparison matrices of features obtained in the previous step were presented, respectively, as input to 15 pre-designed and selected Sci-learn ML models (Table 3). 42 A 7-fold cross-validation process was employed for data discrimination. The 7 folds were chosen based on the number of EEG recordings per class.

**Table 3:** Classifiers used in the present study

Classifier	Hyperparameters
AdaBoostClassifier	Default parameters
BaggingClassifier	Default parameters
DecisionTreeClassifier	Default parameters
ExtraTreesClassifier	Default parameters
GaussianNB	Default parameters
GaussianProcessClassifier	Default parameters
GradientBoostingClassifier	Default parameters
KNearestNeighborsClassifier	Default parameters
LinearDiscriminantAnalysis	Default parameters
LogisticRegression	Default parameters
LogisticRegressionCV	Default parameters
MLPClassifier	Default parameters
RandomForestClassifier	Default parameters
SGDClassifier	Default parameters
Support-vector machines	*γ*: “auto”

The model’s performance was evaluated using a classification report with the following metrics:^43^

- Accuracy: It is calculated as 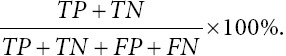. Accuracy represents the overall proportion of correct predictions made by the model. Here, *TP* (true positive) refers to predictions that are positive and actually positive, *TN* (true negative) refers to predictions that are negative and actually negative, *FP* (false positive) refers to predictions that are positive but actually negative, and *FN* (false negative) refers to predictions that are negative but actually positive.
- Recall: It is computed as 
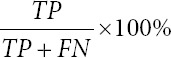
. Recall indicates the proportion of actual positive cases that the model correctly identified.
- Precision: It is calculated using the formula 
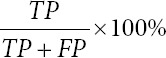
. Precision measures the proportion of positive predictions that the model correctly identified.
- F1-score: It is calculated using the formula 2 
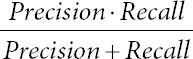
. The F1-score is the harmonic mean of precision and recall.
- Area under the curve (AUC): The AUC of the receiver operating characteristic curve is calculated using the formula 
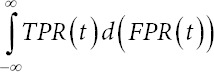
, where *TPR* is the *TP* rate, *FPR* is the *FP* rate and *t* represents each classified instance. AUC measures the model’s ability to distinguish between classes.

## 3. Results

**Figure 2:**
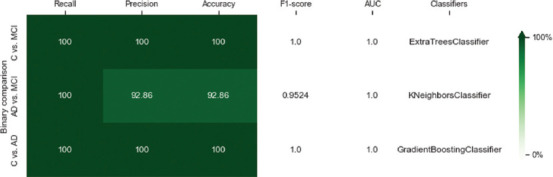
Heatmap of the final report classification results Abbreviations: AD: Alzheimer’s disease; AUC: Area under the curve; C: Healthy controls; MCI: Mild cognitive impairment.

Figure 2 illustrates the discrimination performance of the top-performing classifiers between group pairs, presented as a heatmap. The heatmap employs a green gradient color bar to visually represent the method’s discrimination power, based on recall, precision, and accuracy in pairwise comparisons. Lighter shades of green correspond to lower discrimination power, while darker shades signify higher discrimination power.

## 4. Discussion

Table 4 compares the classification metrics obtained in the present study with those reported in state-of-the-art research. The present study stands out by achieving the highest accuracy and recall across most comparison pairs, with minimal underperformance in the AD versus MCI pair.

**Table 4:** Comparison of the final classification results with state-of-the-art research

C vs. MCI				AD vs. MCI				C vs. AD			
		
Authors	Rec	Spec	Acc	Authors	Rec	Spec	Acc	Authors	Rec	Spec	Acc
Akrofi *et al*.^44^	-	-	90	Buscema *et al*.^45^	89	95	92	Huang *et al*.^46^	90	75	84
Aghajani *et al*.^47^	75	94	84	Cunha *et al*.^48^	-	-	89.7	Mellisant *et al*.^49^	93	95	94
Khatun *et al*.^50^	85	95	88	Huang *et al*.^46^	87	68	78	Petrosian *et al*.^51^	80	100	90
Pirrone *et al*.^52^	-	-	98	Poil *et al*.^53^	88	82	85	Cunha *et al*.^48^	-	-	81.4
Vialatte *et al*.^54^	-	-	93	Pirrone *et al*.^52^	-	-	93.8	Kulkarni55	92	96	94
Cunha *et al*.^48^	-	-	85.5	Rodrigues *et al*.^56^	92	96	94	Pirrone *et al*.^52^	-	-	95.9
Dauwels *et al*.^57^	-	-	83	Araujo *et al*.^58^	-	-	88.9	Tran *et al*.^59^	-	-	91
Rodrigues *et al*.^56^	100	97	98	Cejnek *et al*.^22^	91	85	88	Rodrigues *et al*.^56^	97	95	96
Araujo *et al*.^58^	-	-	78.9	Present work	100	92.8	92.8	Araujo *et al*.^58^	-	-	81
Present work	100	100	100					Zheng *et al*.^16^	96.4	97.4	95.8
								Dogan *et al*.^60^	97.7	84.0	92.0
								Present work	100	100	100

Abbreviations: Acc: Accuracy; AD: Alzheimer’s disease; C: Healthy controls; MCI: Mild cognitive impairment; Rec: Recall; Spec: Specificity; vs.: Versus.

In particular, the study demonstrated superior performance of our strategy compared to existing methods, achieving 100% accuracy in both the C versus MCI and C versus AD classifications. These results represent a 2% improvement in accuracy for C versus MCI and a 4% improvement for C versus AD over the state-of-the-art studies.

However, in the AD versus MCI classification, our study showed a slight underperformance in accuracy, with a 1% lower accuracy compared to the finding of Pirrone *et al.*,52 and a 1.2% lower accuracy compared to the results of Rodrigues *et al*.56 and Buscema *et al*.45 Despite this, it is important to highlight that the present study outperformed prior work by Cejnek *et al*.22 on the same database, with a significant 4.8% improvement in accuracy for the AD versus MCI comparison. Although the present study used the same dataset as Cejnek *et al.*,22 we balanced the dataset in our study by randomly selecting seven EEG recordings from the MCI group in the original dataset. Therefore, comparisons between the two studies should be made with caution. Moreover, it should be noted that the MCI versus AD comparison is the only one to show accuracy results below 100% (92.86%). Patient age may influence these results, as the MCI and AD patients in this study are relatively younger (~70 years) compared to those in other state-of-the-art studies (e.g., 22,45 and 56), which could slightly complicate the discrimination process.

Despite the minor underperformance in AD versus MCI, the overall results of the present study are promising and represent substantial advancements in classification accuracy.

## 5. Conclusion

This study highlights the critical role of EEG in identifying distinct brain activity patterns in patients with AD in early and moderate stages. We trained an EEG-based ML model to differentiate between C, MCI, and AD. Utilizing a balanced EEG dataset, which included seven recordings from each class, we computed 43 time-frequency features from each 1-s segment of EEG data. These features were then compressed using 10 statistical measures, and 15 classifiers were applied within a 7-fold cross-validation framework to classify the different stages.

The algorithm performed well, achieving a 100% accuracy in both the C versus MCI and C versus AD classifications, representing a 2% and 4% improvement over existing methods, respectively. In addition, it achieved a 4.8% improvement in AD versus MCI classification on the same dataset. However, it showed a slight 1.2% decrease in accuracy compared to the best-performing previous studies in AD versus MCI. These results underscore the potential of EEG for early AD diagnosis. Nevertheless, for more reliable generalization, future work should focus on a fair comparison with the state-of-the-art methods that use larger databases and align more closely with real-world clinical applications. Specifically, future studies should aim to enhance results using a larger population and applying the hold-out method (e.g., using 80% of the data for training and 20% for testing) instead of a cross-validation approach. This would help address the issue of unbalanced datasets. Techniques such as data augmentation or using a larger, more balanced dataset could eliminate the need for undersampling, enhance model performance by providing a more representative dataset, and mitigate risks of model bias and overfitting. These strategies would improve generalization and robustness when applied to unseen data in future work using a train-test (hold-out) validation approach.^61^,^62^ Ultimately, future studies should also explore different sliding window strategies (e.g., 25% or 50% overlap) for data analysis, as this may improve the feature extraction process by capturing more continuous information. This approach would provide a more realistic assessment of the algorithm’s performance, particularly for clinical applications.

## Data Availability

The data supporting the findings of this study are available on request from the corresponding author.
